# Assessing Older Adults’ Intentions to Use a Smartphone: Using the Meta–Unified Theory of the Acceptance and Use of Technology

**DOI:** 10.3390/ijerph19095403

**Published:** 2022-04-28

**Authors:** Cheng-Chia Yang, Cheng-Lun Li, Te-Feng Yeh, Yu-Chia Chang

**Affiliations:** 1 Department of Healthcare Administration, Asia University, Taichung 41354, Taiwan; chengchia@asia.edu.tw; 2 Department of Medical Research, Jen-Ai Hospital, Taichung 41265, Taiwan; jah5696@mail.jah.org.tw; 3 Department of Healthcare Administration, Central Taiwan University of Science and Technology, Taichung 40601, Taiwan; tfyeh@ctust.edu.tw; 4 Department of Long Term Care, College of Health and Nursing, National Quemoy University, Kinmen County 892009, Taiwan

**Keywords:** meta-UTAUT, older adults, behavioral intention, smartphone

## Abstract

Barriers to smartphone use often exist among older adults, and increasing smartphone use is beneficial to increasing older adults’ quality of life. Studies of older adults’ smartphone use intentions have mostly adopted the technology acceptance model or unified theory of acceptance and use of technology (UTAUT). However, these models have their limitations. A meta-UTAUT has been developed, but it has not been extensively verified with older adults. This study used the meta-UTAUT model to explore the influences on older adults’ smartphone use intentions and behaviors. A total of 311 adults aged 60 to 75 years who had minimal experience with smartphones were recruited. They participated in a 16 h smartphone training and then completed a questionnaire. The results demonstrated that the meta-UTAUT model can predict older adults’ smartphone use intentions and behaviors. Performance expectancy (PE) and social influence significantly influenced behavioral intention (BI) and attitude toward using smartphones (AT). PE was the strongest factor influencing BI. AT also affected BI. Although facilitating conditions did not significantly affect BI, they had a high influence on AT. To increase smartphone use among older adults, training can be implemented to teach smartphone skills and emphasize the benefits of using smartphones.

## 1. Introduction

Barriers to information technology use often exist among older adults [1]. These barriers include fear, lack of knowledge, and worry that the technology is too complex, resulting in resistance to use [2,3]. Resistance to use may also be due to physical disabilities, declined cognitive state, and a lack of communication skills [4]. Previous studies have stereotypically described older adults as less interested in or having implementing barriers to information technology use [5]. In fact, older adults are capable of learning and innovation when using new technologies [5]. According to Fernández-Ardèvol and Arroyo, older adults were classified as assisted users, basic mobile users, intermediate mobile users, and expert mobile users based on different technological capabilities [6]. In these categories, only assisted users need long-term support to use smartphones, whereas the use of information technology by other categories of users may be affected by individual interests, values, habits, usage, and personal information literacy [7]. Therefore, for older adults, it is a challenging task for stakeholders (such as smartphone manufacturers and application developers) to understand their needs.

Recent studies have indicated that smartphones are conducive to disease control and increasing quality of life [8,9]. Smartphones also increase older adults’ interactions with their social groups [10,11]. In addition, they can satisfy the daily needs of older adults and resolve predicaments generated from physiological barriers caused by aging. This is beneficial to increasing the sense of wellbeing and quality of life in old age [12,13]. Therefore, studying older adults’ smartphone usage behavior is critical [14].

Most studies researching older adults’ behavioral intention (BI) of using smartphones adopted the technology acceptance model (TAM) or unified theory of acceptance and use of technology (UTAUT) as the research framework [15,16,17]. However, these models have their limitations [18]. Specifically, the models ignore the personal characteristics of the participants, which may affect the tendency to use technology, and only consider users’ cognition of information systems. During the aging process, older adults may experience various unfavorable conditions and must adjust to numerous physiological, psychological, and social changes, such as the decline of cognitive functions and the lack of social emotions [19]. Compared to younger generations, older adults’ use of technology is influenced by numerous additional factors. Dwivedi et al. modified the TAM to propose the meta-UTAUT model and identified attitude as a critical factor between the four exogenous variables of UTAUT and BI [20,21]. The present study posits that older adults’ attitudes toward smartphones are the determining factor of their intention to use. Studies have also shown that attitude towards smartphones is more effective than the perceived ease of use and perceived accessibility of smartphones when evaluating older adults’ smartphone use intention [3,21,22,23].

Technology usage behavior and intention are affected by culture, situations, and individual characteristics [17]. Previous studies have mentioned that the educational level of older adults and their willingness to learn new technologies will affect their intention to use smartphones [6]. Accordingly, factors related to technology usage behavior in older adults are influenced by environmental factors and personal characteristics, thereby necessitating further research. Other previous studies mostly used random sampling to discuss smartphone use intention [3,16,24,25]. This research design overlooks the differences in individuals’ knowledge and functional use of smartphones. The present study recruited adults aged 60 years or older as research participants. The inclusion criterion was having insufficient experience with smartphones. A 16 h smartphone training was implemented to provide participants with an equal basis in their knowledge of smartphones and smartphone usage ability. Subsequently, the meta-UTAUT model, a model that has not been widely verified in the older adult population, was employed to analyze the influences on older adults’ smartphone use intentions and behaviors. The results of this study can be applied to develop concrete suggestions to increase technology usage among older adults.

## 2. Theoretical Background and Hypotheses Development

### 2.1. Unified Theory of Acceptance and Use of Technology

Venkatesh et al. integrated eight theories and proposed the UTAUT [26]. The UTAUT has four determining factors: (1) Performance expectancy (PE), such as perceived usefulness. (2) Effort expectancy (EE), or expected amount of work involved. EE is defined as the difficulty of using a system, such as perceived ease of use, system complexity, and actual ease of use of the system. (3) Social influence (SI), which describes the level to which an individual’s important others, such as family and friends, think that they should use a technology. SI involves subjective norms, social factors, and public image. (4) Facilitating conditions (FCs), which describe the degree to which the infrastructure supports the use of information technology, such as perceived behavioral control. Venkatesh et al. discovered that FCs have no direct influence on users’ BI but have a direct influence on users’ behavior [26]. The UTAUT uses gender, age, experience with the technology, and voluntariness as moderating variables, and observes whether they influence the four determining factors and BI.

The UTAUT model has been widely adopted in various disciplines and remains popular. Using the UTAUT to predict the BI of older adults using smartphones has yielded favorable results [15,16,17]. Gao, Yang, and Krogstie used the UTAUT to study factors affecting Chinese older adults’ use of smartphones [27]. The results revealed that 77% of BI can be explained by the model. Studies have also reported that PE and SI significantly affect older adults’ use of smartphones. However, EE was not shown to significantly affect users’ intentions [15,16]. Furthermore, FCs are highly correlated with users’ intention and usage behavior [15,16]. However, Hoque and Sorwar obtained different research results [17]. They reported that FCs did not significantly affect the usage behavior and intention to use technology in older adults, possibly because of the culture of the country where the study was conducted. The study stated that because older adults commonly rely on their children for support, they overlook the importance of technology, money, or their facility and resource needs [17]. Therefore, factors affecting older adults’ use of technology may be affected by personal and environmental characteristics, and further studies are warranted.

### 2.2. Meta-UTAUT

Tamilmani et al. stated that the UTAUT has limitations [18]. Specifically, it does not include personal characteristics that may influence a person to use technology. Dwivedi et al. used meta-analysis and structural equation modeling to reinterpret the UTAUT model [20,21]. They invented the meta-analysis-based UTAUT (meta-UTAUT) and determined that attitude plays a partial mediating effect between the four exogenous variables of UTAUT and BI. Dwivedi et al. noted that a model including attitude can increase the explanatory power for BI. That model used attitude as the mediating variable to create the meta-UTAUT. The meta-UTAUT is both more comprehensive and simpler than the UTAUT [28]. Therefore, this study deemed that the meta-UTAUT model, which has yet to be verified among older adults, could be used to predict the smartphone usage of older adults. Accordingly, this study used the meta-UTAUT model as the basis to explore older adults’ smartphone use intention.

### 2.3. PE and EE

Studies have verified that EE significantly affects use intention [17,29,30,31,32]. In TAM, PE has a strong predictive power for use intention [33]. EE assesses the effort required by users to use a technology [26,33]. Studies have discovered that, for older adults, the barrier in using technology lies in the ease of use of that technology [2,34]. Studies have also verified that the EE for using a technology for older adults affects their use intention [16,17,32,35,36]. Therefore, this study made the following hypotheses:

**Hypothesis** **1** **(H1):** *PE positively affects older adults’ AT toward smartphones.*

**Hypothesis** **2** **(H2):** *EE positively affects older adults’ AT toward smartphones.*

In the TAM, the two most critical factors affecting technology use intention and behavior are perceived usefulness (PU) and perceived ease of use (PEOU) [37]. PU is the degree to which an individual perceives using the technology can improve their quality of life. PEOU is defined as the amount of effort an individual thinks using a technology will involve. Through the attitude toward using the technology (AT), PU and PEOU directly or indirectly predict usage behavior [38]. In the TAM, PE and EE are PU and PEOU, respectively. Past studies have proven the direct relationship between PU and AT [39,40]. Similarly, the influence of PEOU on AT has been verified in different scenarios [41,42]. On the basis of the aforementioned theories, this study proposed the following hypotheses:

**Hypothesis** **3** **(H3):** *PE positively affects older adults’ BI of using smartphones.*

**Hypothesis** **4** **(H4):** *EE positively affects older adults’ BI of using smartphones.*

### 2.4. Social Influence

When assessing the acceptance of technology, SI cannot be overlooked. If the social environment is conducive to using a technology, then this conduciveness plays a critical role in the process of decision making [43]. SI consists of subjective norms, social factors, and image; it is used to assess the degree of support from a users’ friends and family in using the information technology [26]. Numerous studies have verified that SI significantly influences technology usage behavior in older adults [11,17,44]. Most people lack relevant information on how to use innovative technology products and services. Therefore, people close to older adults have a critical influence on personal AT [23,45]. For example, Blok et al. noted that most older adults cannot use a technology product (such as smartphones) by themselves and require assistance from family members or caregivers [44]. Therefore, if people around the older adults forbid the older adults from using technology products, their use intention and behavior will be affected [44]. The present study maintained that older adults did not have the need to use information technology in the past. However, upon discovering that all their family members and friends use smartphones, their BI to use smartphones to maintain interpersonal relationships will be affected. Therefore, SI plays a critical role in use intention and AT [45,46]. On the basis of the aforementioned theories, this study proposed the following hypotheses:

**Hypothesis** **5** **(H5):** *SI positively affects older adults’ AT with smartphones.*

**Hypothesis** **6** **(H6):** *SI positively affects older adults’ BI of using smartphones.*

### 2.5. Facilitating Conditions

FCs reflect the degree to which a user judges that they have the personal resources, professional knowledge, and support necessary to use a technology. They affect whether the user perceives the task as easy or difficult. In the present study, FCs included skills training, information provision, and user support; these FCs affect older adults’ use of smartphones. Studies have listed FCs as a critical factor affecting older adults’ use of technology, such as tablets, smartphones, and wearable devices [15,16,47,48,49,50]. Older adults with sufficient resources to use smartphones (such as the knowledge of use and guidance and support from experts) will perceive that using the technology is easy. These FCs positively influence older adults’ AT [51], and are conducive to increasing use intention [47] (Chen and Chan, 2014). In addition, having higher degrees of FCs, such as having technology support or being taught related knowledge, reduces the effort required to use a technology [49,50]. That is, when the user has sufficient supporting resources, such as the knowledge of using said technology or having technology experts’ guidance and support, they may think that using that technology is simple and easy, and they may desire to use it more. Therefore, this study proposed the following hypotheses:

**Hypothesis** **7** **(H7):** *FCs positively influence older adults’ AT with smartphones.*

**Hypothesis** **8** **(H8):** *FCs positively influence older adults’ BI of using smartphones.*

### 2.6. Attitude toward Using Technology

In the TAM, Davis noted that AT can effectively predict BI [37]. AT is conceptualized as a personal feeling; it can directly or indirectly influence whether a specific behavior is performed. When a person holds a certain AT towards a specific behavior, the person will be more inclined to perform said behavior [52]. According to TAM, the key factor affecting use intention is the AT one holds [37]. This AT refers to the degree of importance one actively or passively places on the technology. Although one study reported that AT and technology use were not significantly correlated [53], a correlation between them was identified in several other studies [22,23,54]. Therefore, we proposed the following hypothesis:

**Hypothesis** **9** **(H9):** *AT positively affects older adults’ BI toward smartphones.*

## 3. Materials and Methods

### 3.1. Smartphone Training Course Procedure

The smartphone training course offered by this study focused on the operation of basic smartphone functions and the use of social, entertainment, and medical applications. The total length of the course was 16 h. Participants were provided sufficient hands-on time to practice using their smartphones during each class. Classes were held twice a week for 4 weeks, with each class lasting 2 h. The course content was divided into four parts: basic knowledge of smartphone operations (4 h), how to use smartphones to socialize (4 h), entertainment functions of smartphones (4 h), and medical applications of smartphones (4 h). All participants were required to bring their own smartphones that used the iOS or Android operating system. In addition to the lecturer, four teaching assistants were arranged for each course. In the course, a teaching assistant led a team (about 5~6 people) to help participants solve operational problems and correct mistakes through group discussions. This teaching design ensures that participants could achieve the objectives of the course. The course was conducted with one device per participant. A total of 3 months was taken to complete the training of all participants. After the participants completed the aforementioned course and the questionnaire survey, they received a TWD 100 coupon for 7-Eleven.

### 3.2. Participants

This study was approved by the Institutional Review Board of Jen-Ai Hospital–Taichung (No. 10927). The study used Taiwan’s community care centers as the channel for recruiting research participants. The researchers visited the community care centers personally to explain the research objectives. Participant recruitment began after the researchers obtained the approval of the responsible persons of the centers. A total of 21 community care centers participated in this study. At each center, we explained the content and purpose of the smartphone training course to the participants. When a participant agreed to join the smartphone training course, it meant they agreed to join this research project. The inclusion criteria were as follows: (1) men and women aged 60 years or older who (2) chose to participate, (3) owned a smartphone with the iOS or Android operating system, and (4) did not know how to use a smartphone fully (such as only using it to make phone calls). The exclusion criterion was having participated in a similar smartphone training course in the past. On average, 15 participants were recruited from each community care center. Ten participants whose attendance was less than 87.5% (1/8) were ultimately excluded. In sum, this study obtained valid data from 311 people.

### 3.3. Data Collection

This study adopted a self-report questionnaire as the data collection tool. The questionnaire consisted of two parts. Part one surveyed the participants’ demographic characteristics: sex, age, education level, and occupation; the duration and frequency of using a smartphone to go online (the frequency of use: every day or nearly every day, at least once a week, at least once a month, less than once a month, or never; and the duration of use: less than 1 h each time, 1–2 h, 2–3 h, 3 h or more, or never); the activities they engage in online (send and receive e-mails, search for information, book hotels or other services, banking, search for health-related information, catch up with the news, shop online, engage in financial activities (such as check stocks), access entertainment (watch videos or play games), use LINE, use Facebook, or do not engage in online activities). Part two of the questionnaire referenced the measurement variables in the meta-UTAUT [28]. PE, EE, SI, FCs, use intention, and AT were used to draft the questionnaire items to capture information about the smartphone usage behavior and intention of older adults. The questionnaire items were tailored to the specific content of this study. All the items were scored using a 5-point Likert scale: strongly disagree (1), disagree (2), neutral (3), agree (4), and strongly agree (5).

### 3.4. Data Analysis

Partial least squares (PLS) was adopted to verify the research hypotheses. PLS was chosen because it can handle a small sample size and avoid the problem of multivariate collinearity. More importantly, it is not affected by limitations concerning data distribution when the sample size is small [55]. Because this study exclusively trained older adults, the participant recruitment process was difficult, resulting in a low sample size. Furthermore, PLS avoids the limitations of variable distribution type and has favorable predictive power and explanatory power. The analysis and explanation of the model using PLS were divided into two stages: (1) assessment of the model reliability and validity; and (2) assessment of the structural model. After verifying that the structure of this study was valid and reliable, we reached conclusions about the correlations in the structure.

## 4. Results

### 4.1. Descriptive Statistics of the Participants

The 311 participants were ranged between 60 and 75 years old with a mean age of 66.2 years old. Most were 60–65 years old (237 people; 76.2%), followed by 66–70 years old (68 people, 21.8%). A total of 179 were women (57.5%), and 132 were men (42.4%). The participants were mostly junior high school graduates (156 people; 50.16%), followed by elementary school graduates (107 people; 34.4%). After receiving the smartphone training, the self-reported smartphone usage frequency and duration of all participants significantly increased. Regarding the duration of use, most participants used a smartphone for 2–3 h per session (158 people; 50.8%), followed by 1–2 h per session (111 people; 35.6%). Regarding the participants’ purpose of using smartphones, the most common reason was to use LINE to communicate with friends and family (83.2%), followed by searching for information, such as medical and health information (77.2%) and accessing entertainment, such as watching videos or playing games (65.8%).

### 4.2. Reliability and Validity of the Research Tool

This study referenced the values of the variance inflation factor (VIF) to prevent high collinearity and determined that a VIF > 10 indicates high collinearity. Each item in this study had a VIF smaller than 10. The standardized root mean squared residual (SRMR) was subsequently used to assess model fit. When the SMRM of the saturated model and the estimated model are both smaller than 0.08, then the model has a favorable model fit. The SRMR values of the saturated model and the estimated model of this study were both 0.06, suggesting that the research model had a favorable model fit. According to Bagozzi and Yi [56], the following three indicators may be used to assess the reflective indicators of the measurement model: individual item reliability, composite reliability (CR), and average variance extracted (AVE) of the latent variables. Individual item reliability is used to assess the factor loading of the measurement variable of a latent variable and to test the statistical significance of the loading of each variable. The factor loadings in this study were all higher than 0.5, which is the recommended value, and were all significant. The sample factor loadings were between 0.831 and 0.935, meeting the recommended criteria for factor loading from Hair et al. [57] (Table 1).

The CR of the latent variables consists of the reliability of all measurement variables. CR reflects the internal consistency of the constructs. A high CR indicates high internal consistency of the latent variables. Chin suggested that the CR should be at least 0.7 [58]. The CRs of this study were between 0.907 and 0.95, indicating that the research model has favorable internal consistency. The AVE of latent variables calculates the explanatory power of the variance of each measurement variable on the latent variable. A high AVE indicates that the latent variable has favorable discriminant validity and convergent validity. Fornell and Larcker suggested that the AVE be at least 0.5 [59]. The AVEs of the latent variables of this study were between 0.764 and 0.852; therefore, the reflective measurement variables of this study had favorable convergent validity. According to Hair et al., the square root of the AVE of latent variables must be greater than the correlation coefficient of other constructs [60]. The square root of the AVE of latent variables of this study met this criterion (Table 2), signifying that the constructs had discriminant validity. The analysis results reveal that the reliability, convergent validity, and discriminant validity of each construct were acceptable.

### 4.3. Verifying Hypotheses

In PLS, bootstrap resampling was conducted to verify the degree of significance of paths in the structural model. *R*^2^ is the main indicator of whether a model is favorable [58]. PLS was used to estimate the correlations of paths between constructs. Standardized coefficients were used as path values. To verify the hypotheses of the path correlations of the research model, we required that the hypotheses reach a significance level of α = 0.05. The path analysis coefficients of the structural model are presented in Table 3.

Except for H8, where FCs did not have a significant influence on BI (β = −0.058, *t* = 0.876; *p* > 0.05), all remaining hypotheses were accepted. PE exhibited a positive effect on AT (β = 0.188, *t* = 2.021; *p* < 0.05); thus, H1 was accepted, revealing that people who had high expectations for the performance of smartphones had a positive attitude towards them. Next, because EE exhibited a positive effect on AT (β = 0.232, *t* = 2.104; *p* < 0.05), H2 was accepted. SI positively influenced AT (β = 0.211, *t* = 3.595; *p* < 0.001), suggesting that older adults’ attitude towards smartphones was influenced by SI; hence, H5 was accepted. Finally, as FC positively affected AT (β = 0.254, *t* = 2.813; *p* < 0.001), H7 was accepted, indicating that when people had more resources and knowledge about smartphones, their attitude toward smartphones was more positive. A total of 60.5% of the variance of AT was explained (*R*^2^ = 0.605).

PE had positive effects on BI (β = 0.513, *t* = 6.056; *p* < 0.001), so H3 was accepted, indicating that when people expected smartphones to perform well, their intention to use them was high. EE positively affected BI (β = 0.217, *t* = 2.327; *p* < 0.01), so H4 was accepted, revealing that EE affected BI. SI positively influenced older adults’ BI of using smartphones (β = 0.106, *t* = 2.099; *p* < 0.05), indicating that, the greater the SI value, the stronger older adults’ intention to use smartphones, so H6 was accepted. Finally, attitude affected BI (β = 0.136; *t* = 2.491; *p* < 0.01), so H9 was accepted.

**Figure 1:**
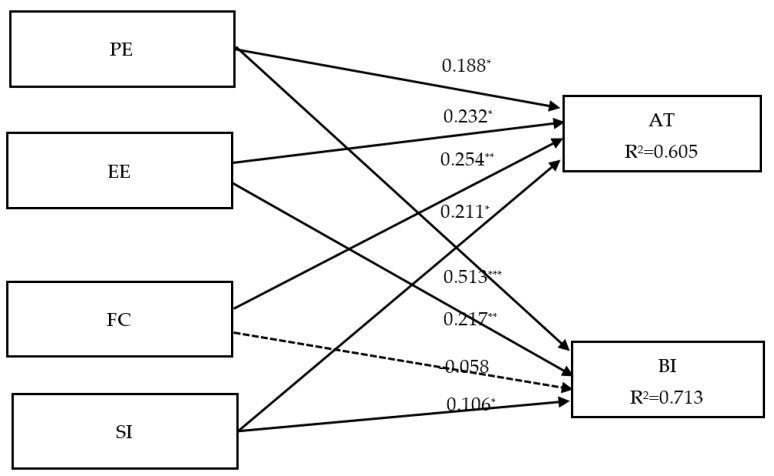
Structural model analysis results. Note: * *p* < 0.05; ** *p* < 0.01; *** *p* < 0.001.

A total of 71.3% of the variance of BI was explained (*R*^2^ = 0.713). This study adopted the *Q*^2^ of Stone–Geisser to assess the predictive relevance of the model. *Q*^2^ > 0 indicates that the model had predictive power [58]. According to Geisser (1974), a larger *Q*^2^ has stronger predictive power (0.02 = small, 0.15 = medium, and 0.35 = immense). In this study, BI exhibited a *Q*^2^ of 0.576, whereas AT exhibited a *Q*^2^ of 0.429, both of which had immense predictive power, indicating that the research model had predictive power (see Figure 1).

## 5. Discussion

This study explored the use intention of smartphones in older adults. This study differed from previous studies that utilized random sampling to survey use intention [3,16,25]. In this study, participants all received a 16 h smartphone education training, which allowed them to have high similarity in their knowledge about smartphones and in their technology use experience. Second, this study used the meta-UTAUT model to understand the relationship between the factors affecting older adults’ acceptance of smartphones. The results indicated that the meta-UTAUT model can explain 71.3% of the variance of BI. These findings were similar to those of previous studies [28,61], which reported that the meta-UTAUT model framework can be used to explain older adults’ usage of smartphones. To our knowledge, this study was the first to use the meta-UTAUT model in older adults. The research results supported the validity of using meta-UTAUT as a theoretical basis. In addition, they proved that AT critically affected older adults’ smartphone use intention. Furthermore, AT and users’ BI were strongly correlated [28,61].

### 5.1. Theoretical Implications

Many studies have stated that AT is not critical in the TAM, even suggesting removing it from the model [53,62,63]. However, a recent study reported that older adults’ technology AT is a factor that predicts BI [3]. The present study also proved that AT is an indispensable predictive factor for older adults using smartphones.

In this study, in addition to AT, older adults’ PE, EE, and SI significantly affected BI and AT toward smartphones. Specifically, their PE had the strongest influence on BI, indicating that, when older adults perceived that smartphones would bring them a high benefit, they had an increased intention to use smartphones. As Mitzner et al. proposed, older adults’ intention to use technology is driven by the perception of benefits [64]. This study also demonstrated that PE had an impact far greater than that of other factors on BI. If older adults cannot perceive the benefits of using smartphones, they will not find this technology beneficial; consequently, this will affect their BI and AT [65].

In TAM, PEOU is a critical factor affecting users’ BI regarding technology. Therefore, understanding the predicaments faced by users in their use of technology is crucial because PE affects BI and AT. Mitzner et al. proved that, as long as older adults find information technology useful and easy to use, they will have the intention to use it [64]. Therefore, more education should be provided to enable older adults to use smartphones to easily complete daily life tasks or access entertainment, such as video conferencing, looking up bus schedules, and ordering take-out food. Through education, this study increased older adults’ knowledge and technology application ability regarding smartphones. Education allowed them to find the technology easy to use, which affected their BI and AT toward smartphones [66,67,68]. This study proved that training is conducive to increasing older adults’ EE and PE, which resulted in a positive effect on older adults’ BI to use smartphones.

However, Guner and Acarturk reported that PU and PEOU in older adults did not significantly affect their BI, which was drastically different from our conclusions [3]. This difference may be due to Guner and Acarturk randomly recruiting their research participants (including retirees and older adults in long-term care facilities), which was different from the sample structure of the present study [3]. Participants of the present study all received 16 h of training on smartphone use. During the process, they might have realized the benefits smartphones can bring to their daily life. Furthermore, during the learning process, they perceived the ease of use of smartphone technology. As a result, their BI was significantly influenced.

Regarding SI, one study discovered that older adults tend to ignore the influences of social stress, images, and social status while pursuing goals with emotive meanings; as a result, SI did not exhibit a significant impact on older adults’ BI toward technology [36]. However, the present study observed that SI significantly affected BI and AT. As Graf-Vlachy, Buhtz, and König noted, when users learn how to use social software, they experience social impulses, which are part of SI [69]. The participants of this study consisted of older adults who participated in community center activities. They were all friends or neighbors from the same community. After the training course, they engaged in numerous online social interactions. This kind of group interaction made SI an impact factor for predicting older adults’ intention to use smartphones.

Choudrie, Pheeraphuttranghkoon, and Davari noted that people are aware that older adults wish to further understand how to use smartphones [25]. However, few are willing to help them. One study discovered that FCs critically influence the BI of technology acceptance [36]. However, in the present study, FCs did not exhibit a significant impact on older adults’ BI of using smartphones. We inferred that, because the participants received the smartphone training course, all of them were equipped with the resources and knowledge to use smartphones. Therefore, they might not have been aware of the importance of skills or knowledge. Consequently, FCs did not affect their smartphone use intention. As Hoque and Sorwar mentioned [16], the factors affecting older adults’ use of technology were mainly PE, EE, and SI, rather than promotive factors such as resource support. Notably, FCs were the most critical antecedent that influenced AT. One study stated that providing FCs, such as supportive services or knowledge, critically affects AT and usage BI [18]. Following the same line of thinking, the present study maintained that FCs were the strongest factor affecting AT.

### 5.2. Practical Implications

The older adult population is exhibiting the fastest growth in smartphone usage. However, whether older adults use smartphones depends on their assessment of whether smartphones benefit their daily life or personal activities. In a digitalized society, older adults must use technology to fulfill their daily necessities and live independently [10,11]. An increasing number of companies have used smartphones to offer a wide variety of support services to this group [9,36,70], allowing them to meet their daily needs without leaving home and solving physiological barriers. Technology can be used to develop and maintain social relationships, thereby increasing older adults’ interests in information technology [71]. To increase older adults’ positive attitudes toward smartphone use, training can be implemented to emphasize the advantages of smartphones. Users also need to be taught how to use smartphones and the benefits of using them, thereby increasing technology acceptance. These methods can reduce older adults’ anxiety about technology [17,72], and are conducive to increasing older adults’ AT, indirectly increasing their use intention. In addition, if the training course includes community peers, family members, and caregivers, social connections can be utilized to promote older adults’ AT and willingness to use smartphones [73]. Finally, smartphones have become an integral part of everyday life, even for older adults [7]. Therefore, it is suggested that stakeholders can take the advice of older adults into consideration when designing smartphone applications related to daily life or developing hardware devices. Stakeholders can even make older adults into early users to reduce the unfriendly situations caused by the design of products [5]. In this way, it is more likely to improve the older adults’ willingness to use technology.

### 5.3. Research Limitations and Future Research Directions

This study has several limitations. First, the sample size of this study is insufficient to represent all older adults. This study adopted purposive sampling to recruit participants from community centers. These communities were located in both metropolitan areas and rural areas. These geographical differences may affect the technology literacy of the participants we recruited, leading to differences in the results from different centers; the participating centers may not have been representative of the entire older population. In addition, the participants recruited in this study all have smartphones, which also limits the range of groups that can be examined. Although these limitations did not affect the research results of this study, we suggest that caution should be taken when interpreting the results. Furthermore, this study did not consider individual differences among older adults (such as gender, education level, or technology literacy level). In the future, model verification can be conducted on these individual differences.

## 6. Conclusions

This study used the meta-UTAUT model to explore the influences on older adults’ behavior and intention of using smartphones. To ensure that the participants had a consistent knowledge level about smartphones, all participants received 16 h of smartphone training courses. The results revealed that the meta-UTAUT model can predict older adults’ smartphone use intention and behavior. PE and SI significantly influenced BI and AT, and were identified as critical predictive factors. PE was the strongest factor influencing BI. AT also affected older adults’ BI of using smartphones. Although FCs did not significantly affect BI, they had a large influence on AT.

## Figures and Tables

**Table 1:** Indicator loading and composite reliability of variables and indicators.

Construct	Item Code	Indicator Loading	Cronbach’s Alpha	CR	AVE	VIF
**Performance expectancy** **(PE)**	PE1	0.866	0.911	0.932	0.79	2.668
	PE2	0.892				3.042
	PE3	0.91				3.529
	PE4	0.888				3.345
**Effort expectancy** **(EE)**	EE1	0.892	0.93	0.95	0.826	3.355
	EE2	0.93				4.377
	EE3	0.893				2.939
	EE4	0.92				3.612
**Facilitating conditions** **(FC)**	FC1	0.899	0.87	0.92	0.794	2.732
	FC2	0.914				2.917
	FC3	0.859				1.884
**Social influence** **(SI)**	SI1	0.831	0.846	0.907	0.764	1.763
	SI2	0.917				2.412
	SI3	0.873				2.243
**Attitude towards using (AT)**	AT1	0.935	0.913	0.945	0.852	3.645
	AT2	0.912				2.762
	AT3	0.922				3.33
**Behavioral intention** **(BI)**	BI1	0.913	0.89	0.932	0.82	2.599
	BI2	0.878				2.400
	BI3	0.925				3.054

**Table 2:** Discriminant validity.

	Mean	S. D.	PE	EE	FC	SI	AT	BI
**PE**	4.196	0.762	**(0.889)**					
**EE**	4.125	0.768	0.843	**(0.909)**				
**FC**	4.083	0.655	0.744	0.826	**(0.891)**			
**SI**	3.880	0.749	0.628	0.586	0.611	**(0.874)**		
**AT**	4.323	0.752	0.632	0.649	0.616	0.481	**(0.923)**	
**BI**	4.35	0.719	0.828	0.774	0.676	0.611	0.596	**(0.905)**

Note 1: PE: performance expectancy; EE: effort expectancy; FC: facilitating conditions; SI: social influence; AT: attitude toward use of the technology; BI: behavioral intention. Note 2: The square root of the AVE values shown in bold represent.

**Table 3:** Effects on endogenous variables.

Effect	Relations	Estimate	SE	T-Value	95% CI LL	95% CI UL
**Direct Effects**						
H1	PE–AT	0.188 *	0.093	2.021	0.030	0.340
H2	EE–AT	0.232 *	0.110	2.104	0.051	0.411
H3	PE–BI	0.513 ***	0.085	6.056	0.376	0.657
H4	EE–BI	0.217 **	0.093	2.327	0.054	0.366
H5	SI –AT	0.211 **	0.090	3.595	0.092	0.392
H6	SI–BI	0.106 *	0.050	2.099	0.023	0.193
H7	FC–AT	0.254 ***	0.059	2.813	0.107	0.306
H8	FC–BI	−0.058	0.066	0.876	−0.173	0.042
H9	AT–BI	0.136 **	0.055	2.491	0.043	0.223
**Control Variables**						
Education → BI		0.037	0.037	1.008	−0.021	0.100
Gender → BI		−0.001	0.039	0.031	−0.069	0.061
Age → BI		−0.009	0.030	0.281	−0.057	0.046

PE: performance expectancy; EE: effort expectancy; SI: social influence; FC: facilitating conditions; BI: behavioral intention; AT: attitude toward use of the technology. * *p* < 0.05; ** *p* < 0.01; *** *p* < 0.001.

## Data Availability

The data that support the findings of this study are available on request from the corresponding author. The data are not publicly available due to privacy or ethical restrictions.
